# Association between Heavy Metals, Metalloids and Metabolic Syndrome: New Insights and Approaches

**DOI:** 10.3390/toxics11080670

**Published:** 2023-08-03

**Authors:** Airton C. Martins, Beatriz Ferrer, Alexey A. Tinkov, Samuel Caito, Romina Deza-Ponzio, Anatoly V. Skalny, Aaron B. Bowman, Michael Aschner

**Affiliations:** 1 Department of Molecular Pharmacology, Albert Einstein College of Medicine, New York, NY 10461, USA; airton.dacunhamartinsjunior@einsteinmed.edu (A.C.M.);; 2 Laboratory of Ecobiomonitoring and Quality Control, Yaroslavl State University, 150003 Yaroslavl, Russia; tinkov.a.a@gmail.com (A.A.T.);; 3 IM Sechenov First Moscow State Medical University (Sechenov University), 119435 Moscow, Russia; 4 School of Pharmacy, Husson University, Bangor, ME 04401, USA; 5 School of Health Sciences, Purdue University, West Lafayette, IN 47907-2051, USA; bowma117@purdue.edu

**Keywords:** arsenic, cadmium, diabetes, hypertension, lead, mercury, obesity

## Abstract

Metabolic syndrome (MetS) is an important public health issue that affects millions of people around the world and is growing to pandemic-like proportions. This syndrome is defined by the World Health Organization (WHO) as a pathologic condition characterized by abdominal obesity, insulin resistance, hypertension, and hyperlipidemia. Moreover, the etiology of MetS is multifactorial, involving many environmental factors, including toxicant exposures. Several studies have associated MetS with heavy metals exposure, which is the focus of this review. Environmental and/or occupational exposure to heavy metals are a major risk, contributing to the development of chronic diseases. Of particular note, toxic metals such as mercury, lead, and cadmium may contribute to the development of MetS by altering oxidative stress, IL-6 signaling, apoptosis, altered lipoprotein metabolism, fluid shear stress and atherosclerosis, and other mechanisms. In this review, we discuss the known and potential roles of heavy metals in MetS etiology as well as potential targeted pathways that are associated with MetS. Furthermore, we describe how new approaches involving proteomic and transcriptome analysis, as well as bioinformatic tools, may help bring about an understanding of the involvement of heavy metals and metalloids in MetS.

## 1. Introduction

Metabolic syndrome (MetS) constitutes a major public health issue due to its increasing worldwide prevalence. Global statistics show that a quarter of the adult population has developed MetS [1]. As stated by the National Heart, Lung, and Blood Institute (NHLBI), the development of MetS requires the presence of three of the following clinical conditions: abdominal obesity, high blood pressure, high triglyceride levels, low HDL cholesterol, and impaired fasting blood glucose [2]. The presence of these symptoms raises the risk of developing stroke, cardiovascular disease, and type 2 diabetes. According to the National Health and Nutrition Examination Survey (NHANES) data, during 2017–2018, the prevalence of MetS was 38.3% in the U.S. adult population [3]. The incidence of MetS parallels the incidence of obesity, the most prevalent outcome of metabolic syndrome. Thus, in 2017–2018, U.S. obesity prevalence was 42.4% [4].

Obesity is a complex multifactorial disease/syndrome characterized by an excess of adiposity concomitant with metabolic alterations. Fundamentally, an imbalance between energy intake and expenditure leads to increased fat mass and obesity. Many factors contribute to weight gain, including genetics, stress, consumption of high-caloric meals, sedentary lifestyles, altered sleep routines, and certain medications [5,6,7,8,9,10]. Recent work has focused on identifying other factors that affect the risk of obesity. New data suggest that environmental exposure factors, such as heavy metals, are also a risk factor for MetS and obesity [11,12,13]. Moreover, some risk factors can be associated with metals and metalloids, even in low-level exposure (i.e., below the governmental limits and guidelines). For example, low levels of arsenic exposure are still associated with obesity in postmenopausal women, most likely due the fact that they have decreased estrogen levels, which increases the circulating concentrations of arsenic (As) [14].

The progress of urbanization and industrialization has dramatically increased metal pollution, including heavy metals and metalloids such as As [15,16]. Heavy metals are naturally existing metallic elements with high atomic weights and high density compared to water. They are used in several industrial processes, such as mining, medical, technological, and agricultural sectors. Once they are released into the environment, they cannot be destroyed or biodegraded. Thus, heavy metals are persistent environmental contaminants. Contaminated water and food consumption, industrial operations, occupational, cigarette smoke, fossil fuel, waste, and cosmetic preparations are sources of heavy metal exposure [17]. Upon exposure, heavy metals bioaccumulate, increasing the risk of chronic health issues [17,18,19].

Several animal and human studies have shown that exposure to heavy metals is associated with MetS and obesity. A cross-sectional epidemiologic study in the Korean population found a positive association between visceral adiposity and blood mercury [20,21]. Similarly, NHANES data from 2003–2014 found an association between obesity and cumulative exposure to heavy metals among U.S. adults [22]. In vivo studies have shown that alterations in adipogenesis, adipocytokines secretion, and the hypothalamic dopaminergic system might act as potential mechanisms that mediate the association between heavy metals and obesity [23,24,25]. These and other studies have highlighted the association between heavy metals and MetS. In this review, we summarize the mechanisms of five of the most harmful heavy metals that may play a role in inducing obesity and MetS. In addition, we uncover different proteomic and transcriptome approaches used to study the association between heavy metals and metabolic syndrome.

### 1.1. Cadmium (Cd)

Recent epidemiological studies demonstrated that Cd exposure may be associated with a risk for MetS, although the relationship of metal body burden with MetS components varies between the studies. Specifically, in a study of 150 individuals, high urinary Cd levels were characterized by increased odds of MetS, being also associated with lower HDL-C levels [26]. The results of a recent cross-sectional study of 140 individuals demonstrated that high serum Cd levels were associated with a 3-fold higher risk of dyslipidemia (OR: 3.05 [95% CI: 1.19–7.86], *p* = 0.02), but not obesity or diabetes [27]. Correspondingly, the results of a meta-analysis of 11 studies confirmed the association between Cd exposure and low HDL-cholesterol and increased triglyceride levels, but not other components of MetS, whereas the association with the risk of MetS was significant only in Asian populations [28]. Another NHANES-based study demonstrated that despite a significant association with MetS and low HDL-C levels, urinary Cd concentration was inversely related to the risk of abdominal obesity [29]. Cd exposure was shown to modulate the association between circulating LPS and MetS. Specifically, high levels of LPS were directly associated with MetS only in men with higher than median blood Cd levels, but not lower Cd exposure, in a study of 200 assumed healthy individuals [30]. Analysis of NHANES 2011–2018 data demonstrated that elevated blood Cd level was associated with lower risk of MetS [31]. Therefore, Cd appears to be differentially associated with particular components of metabolic syndrome.

Strong evidence for a role of Cd in hypertension exists, although the association varies significantly for different Cd exposure biomarkers used and specific cohorts of the studied subjects [32]. For example, urinary Cd level was found to be associated with increased risk of hypertension, whereas blood Cd was associated with elevated systolic and diastolic blood pressure only in Non-Hispanic black Mexican-American women using NHANES data [33]. An examination of subjects from the ESTEBAN survey (2014–2015) demonstrated a significant association between urinary Cd and hypertension only in subjects with obesity and chronic kidney function [34]. The results of NHANES (1999–2010) demonstrated that blood Cd levels were positively associated with both SBP and DBP, whereas urinary Cd concentration was characterized by a direct and inverse relationship with DBP and SBP, respectively. In addition, positive association between urinary Cd and SBP was revealed in subjects with moderate or severe kidney dysfunction [35]. The results of a prospective cohort study of just over 3000 American Indians demonstrated that higher baseline urinary Cd level was associated with higher increase in both systolic (+0.62 (0.37–0.87) mm Hg) and diastolic (+0.18 (0.05–0.31) mm Hg) blood pressure, as well as increased risk of hypertension [36].

The association between Cd exposure and hypertension risk was also demonstrated in meta-analysis studies. Specifically, the results of a recently published meta-analysis demonstrated a positive relationship between both blood and hair Cd levels and hypertension risk [37]. An earlier meta-analysis also revealed a significant positive relationship between occupational Cd exposure and hypertension risk [38]. Taken together, epidemiological findings are strongly indicative of the role of Cd exposure as a risk factor for hypertension.

**Figure 1:**
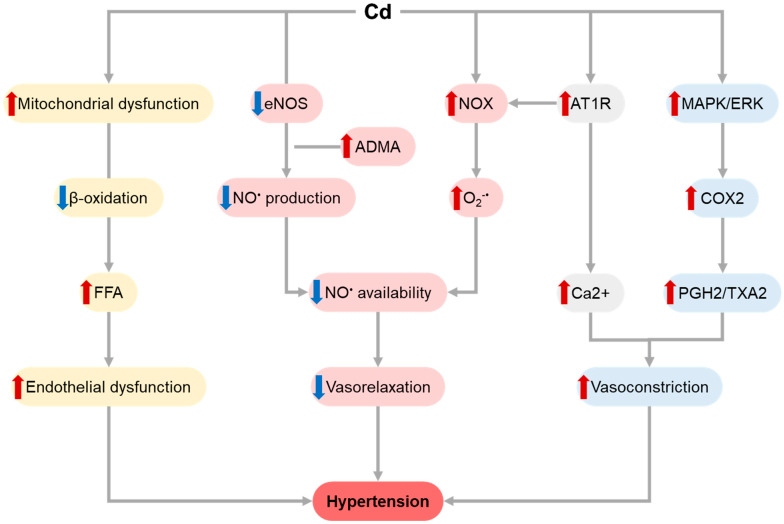
Molecular mechanisms involved in atherogenic effects of Cd exposure. Cd-induced ROS overproduction promotes LDL oxidation and oxLDL internalization by macrophages, resulting in foam cell formation. LDL oxidation is also aggravated by Cd-induced PON1 inhibition. Up-regulated expression of adhesion molecules including ICAM1 and VCAM1 following Cd exposure increases monocyte adhesion and infiltration with their subsequent transformation to foam cells. Proinflammatory effect of Cd also contributes to atherogenesis through promotion of TMAO-induced NF-κB and subsequent NLRP3 inflammasome activation, as well as JAK2/STAT3-dependent M1 macrophage (Mφ) polarization. Finally, Cd-induced alterations of gut microbiota composition along with increased gut wall permeability results in an increase in circulating LPS levels, also promoting proinflammatory signaling. Different colors (pink, grey, blue, yellow) are indicative of distinct pathways for better visualization. Red and blue arrows show the increase and decrease effects.

The molecular mechanisms of the hypertensive effects of Cd have been extensively studied, demonstrating the critical role of impaired NO signaling [32] (Figure 1). Recent studies further addressed the role of Cd in altered NO metabolism, demonstrating that along with promotion of atherogenic phenotype in ApoE−/− mice, Cd exposure significantly affected vascular reactivity, decreasing acetylcholine-induced vasodilatation in aortic rings at least partially due to the inhibition of NO bioavailability [39]. The latter may be mediated by Cd-induced up-regulation of NADPH-oxidase-mediated superoxide production and subsequent peroxynitrite formation. In addition, the hypertensive effect of Cd exposure was associated with up-regulation of angiotensin II type 1 (AT1) receptor expression [40]. Altered vascular response to acetylcholine was shown to be associated with Cd-induced increase in asymmetric dimethylarginine bioavailability [41].

NO production by endothelial cells exposed to non-toxic Cd levels may be associated with impaired TCA cycle, mitochondrial dysfunction, and inflammatory response [42], as well as inhibition of endothelial NOS protein expression [43] and phosphorylation [43]. In addition to reduced production of vasodilator NO, Cd increased COX-2-mediated production of vasoconstrictor thromboxane A2 and prostaglandin H2 [44]. In addition to the balance between vasorelaxation and vasoconstriction factors, Cd was also shown to interfere with the central mechanisms of vascular tone regulation [45]

Endothelial dysfunction also significantly contributes to the hypertensive effects of Cd [46]. Cd promoted TG decomposition along with inhibition of fatty acid oxidation, resulting in an overaccumulation of toxic FFAs that induced endothelial dysfunction through modulation of ROS production and mitochondrial dysfunction [47]. Along with the prooxidant effect of the metal [48], increased p38-MAPK and ERK signaling was shown to underlie Cd-induced endothelial dysfunction and apoptosis [49].

Although it has been proposed that Cd is a risk factor for obesity [50], epidemiological data addressing this association are rather contradictory, demonstrating both positive and negative relationships [51]. The latter has been confirmed by recent observations demonstrating an inverse association between blood Cd and obesity in adults, from NHANES (1999–2014) [33]. Despite a direct relationship between increased blood Cd levels and the prevalence of prediabetes, its association with the prevalence of overweight and obesity was found to be inverse in adults from a cross-sectional SPECT-China study [52]. It is notable that high adipose tissue Cd content was associated with elevated circulating insulin levels and insulin resistance, as well as DM2 risk in a study of 132 current smokers with serum samples [53], although BMI and, specifically, obesity negatively correlated with adipose tissue Cd level from the same study [54].

Recent studies also addressed the impact of Cd exposure on childhood and adolescent obesity. Specifically, maternal blood Cd levels were found to be significantly associated with increased risk of juvenile obesity in children from a Newborn Epigenetics Study (NEST) [55]. In contrast, an inverse association between maternal urinary Cd levels and adolescent adiposity in the offspring was observed, especially in females [56]. In addition, urinary Cd levels were inversely associated (OR 0.46; 95% CI 0.33–0.64; *p* < 0.001) with obesity in 6–19 years old children [57].

Taken together, the existing epidemiological data do not support the role of Cd exposure as a risk factor of obesity. However, accumulation of Cd in adipose tissue as well as reduced odds of overweight and obesity upon Cd exposure demonstrate that adipose tissue appears to be a target for Cd toxicity, affecting its functioning [58]. Transcriptomic analysis demonstrated that Cd exposure results in significant alterations of genes involved in multiple pathways, including adipogenesis, lipid metabolism, insulin response, trace element homeostasis, and inflammation [59].

The toxic effects of Cd on adipose tissue were shown to include alterations in adipogenesis, lipid metabolism, adipokine dysregulation, and inflammation [58]. Cd exposure was shown to accumulate in WAT and reduce adipocyte adiponectin and leptin mRNA and protein expression, being indicative of the role of adipose tissue as a target for Cd toxicity [60]. These findings corroborate earlier observations that Cd exposure inhibited adipogenesis by down-regulation of CCAAT/enhancer-binding protein alpha (C/EBPα) and peroxisome proliferator-activator receptor gamma (PPARγ) protein expression in a dose-dependent manner, also resulting in reduced adipocyte size and inhibition of adipocyte-specific adiponectin and resistin mRNA expression [61,62]. The reduction in body adiposity following Cd exposure was also associated with down-regulation of hypothalamic LepR expression, along with other alterations in the hypothalamic–pituitary–gonadal (HPG) axis [63].

Several studies have demonstrated the propensity of Cd to promote adipose tissue hypertrophy and dysfunction. Specifically, exposure to Cd at clinically relevant levels significantly increased adipose tissue mass and induced insulin resistance in lean mice [64]. In bone marrow-derived mesenchymal stromal cells, Cd exposure induced a shift from osteogenesis to adipogenesis through the up-regulation of PPARγ expression [65,66]. Increased adiposity following early-life Cd exposure in male mice was posited to be mediated by alterations in gut microbiota characterized by reduced biodiversity and increased relative abundance of *Bacteroidetes* and a reduction in *Firmicutes* abundance at the phylum level. At the genus level, Cd exposure induced a reduction in carbohydrate-utilizing *Bifidobacterium* and *Prevotella* in parallel with increased relative abundance of Cd-accumulating *Sphingomonas* [67].

The role of Cd as a causal factor in diabetes has been addressed [68]. Our own previous meta-analysis demonstrated that Cd exposure is associated with higher odds for prevalence of prediabetes (1.60 (95% CI 1.25 to 2.06)) and (1.04 (95% CI 0.99 to 1.10)), as well as increased risk of diabetes (1.38 (95% CI 1.12 to 1.71)) [51]. The most recent epidemiological studies further highlighted the association between Cd exposure biomarkers and the risk of DM2. Specifically, a longitudinal prospective Wuhan-Zhuhai cohort study demonstrated that during three-year follow-up, urinary Cd levels were associated with hyperglycemia and DM2 risk [69]. The association between urinary Cd and DM2 was greater in subjects with high-circulating CRP levels, being indicative of the role of inflammation in the relationship between Cd exposure and DM risk [70]. The association between Cd exposure and prediabetes was evident in overweight and obese males, but not females [71].

Results of a dose–response meta-analysis demonstrated that urinary Cd levels were positively associated with DM and every 1 μg/g creatinine increase in urinary Cd is associated with a 16% higher risk of DM, being in agreement with earlier findings [72,73]. Filippini et al. (2022) also demonstrated a linear association between Cd exposure and DM risk that increases gradually at blood Cd levels exceeding 1 µg/L, whereas prediabetes risk increased only up to 2 µg Cd/g of creatinine in urine, subsequently reaching a plateau at higher concentrations in a meta-study of 42 eligible studies [74]. At the same time, a recent prisma-compliant systematic review and meta-analysis demonstrated that neither blood nor urinary Cd levels were associated with DM2 risk [75].

The observed insulin resistance and diabetes in Cd-exposed subjects was shown to be secondary to Cd’s ability to alter both the insulin signaling cascade and induce β-cell dysfunction and impaired insulin secretion [51,76]. Recent findings significantly expanded the understanding of molecular mechanisms underlying diabetogenic effect of Cd exposure.

Pancreatic islet was characterized as having the highest accumulation of Cd (vs whole pancreas or renal cortex) [77]. Hong et al. (2022) demonstrated that Cd suppressed insulin production and pancreatic β-cell viability through induction of mitochondrial dysfunction associated with mitoROS overproduction and subsequent overproduction of proinflammatory IL-1β, IL-6, and TNF-α [78]. The latter may be mediated by Cd-induced activation of NF-kB signaling in the pancreas [79]. Correspondingly, Cd-induced beta cell damage was also mediated by ferroptosis, characterized by increased iron accumulation, GSH depletion, and inhibition of Gpx4 activity in parallel with proinflammatory signaling via the Ager/Pkc/p65 pathway [80]. Inhibition of STAT6 signaling, as well as a shift to Th1-mediated immune response, along with endoplasmic reticulum stress may be also responsible for pancreatic beta cell apoptosis [81,82]. Apoptosis in beta cells was also induced by Cd via Ca2+-dependent JNK activation and subsequent C/EBP homologous protein (CHOP) signaling [83].

Accumulation of Cd in pancreatic Langherhans islets was also associated with transcriptomic alterations characterized by increased expression of Synj2, Gjb1, Rbpjl, Try5, and 5430419D17Rik genes following in vivo exposure, and up-regulated expression of Mt1, Sphk1, Nrcam, L3mbtl2, Rnf216, and Itpr1 gene expression upon ex vivo exposure, with altered expression of Rbpjl, Mt1, and Itpr1 being potentially associated with altered glucose metabolism [84]. Metabolomic analysis demonstrated that Cd exposure disrupts mitochondrial TCA and fatty acid oxidation in pancreatic β-cells.

Cd-induced insulin resistance was shown in rodent models to be mediated by increased phosphorylation of insulin receptor at threonine 1375 and IRS at serine 308, up-regulation of ERK1/2 and S6K expression, and inhibition of Akt phosphorylation at serine 473, altogether resulting in impaired insulin signaling [85,86]. Prenatal Cd exposure was also shown in rodent models (mouse and rat) to result in offspring insulin resistance, hyperglycemia, and impaired glucose tolerance in adulthood due to up-regulated gluconeogenic p-CREB, PGC-1α, and G6PC protein expression [87,88].

Our previous meta-analysis demonstrated that Cd exposure was associated with higher circulating TC levels (OR = 1.48, 95% CI: 1.10–2.01) and LDL-C levels (OR = 1.31, 95% CI 0.99–1.73), while being inversely related to lower HDL-C concentrations (OR = 1.96, 95% CI: 1.09–3.55) [89]. However, several recent studies failed to reveal any significant association between Cd exposure biomarkers and atherosclerosis risk or dyslipidemia. Being in agreement with indications of the relationship between Cd exposure and atherogenic lipid profile, Cd overload is considered as a risk factor of atherosclerosis [90,91,92]. The results of a dose–response analysis demonstrated that Cd overexposure was associated with a more than twofold increase in the risk of subclinical lower extremity atherosclerosis, that increased monotonically at blood Cd levels >0.69 μg/L [93]. Correspondingly, high blood Cd levels (0.39 to 8.5 μg/L with a median of 0.63 μg/L) are associated with the prevalence of coronary artery atherosclerosis, as evidenced by coronary artery calcium score in a population with low to moderate cadmium exposure [94].

**Figure 2:**
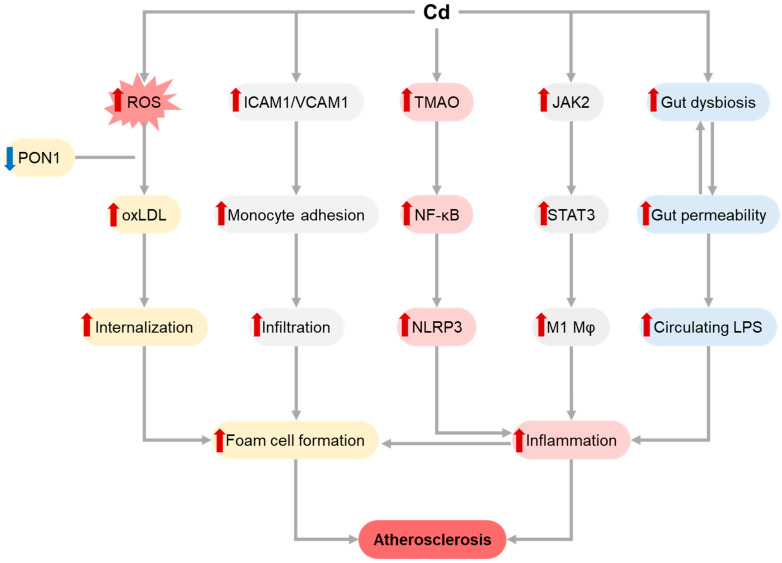
Molecular mechanisms involved in atherogenic effects of Cd exposure. Cd-induced ROS overproduction promotes LDL oxidation and oxLDL internalization by macrophages, resulting in foam cell formation. LDL oxidation is also aggravated by Cd-induced PON1 inhibition. Up-regulated expression of adhesion molecules including ICAM1 and VCAM1 following Cd exposure increases monocyte adhesion and infiltration, with their subsequent transformation to foam cells. Proinflammatory effect of Cd also contributes to atherogenesis through promotion of TMAO-induced NF-κB and subsequent NLRP3 inflammasome activation, as well as JAK2/STAT3-dependent M1 macrophage (Mφ) polarization. Finally, Cd-induced alterations of gut microbiota composition along with increased gut wall permeability results in an increase in circulating LPS levels, also promoting proinflammatory signaling. Different colors (pink, grey, blue, yellow) are indicative of distinct pathways for better visualization. Red and blue arrows show the increase and decrease effects.

In addition to increased lipid biosynthesis and its oxidation due to Cd-induced ROS overproduction [89], Cd has been shown to induce internalization of LDLs and oxLDLs into macrophages, thus promoting foam cell formation [95]. Cd exposure increased 35S-sulfate incorporation and LDL binding affinity of carotid artery proteoglycans, resulting in subendothelial retention of atherogenic lipoproteins [96]. The atherogenic effects of Cd exposure may also involve inhibition of paraoxonase 1 activity [97]. Figure 2 illustrates the proposed mechanisms involved in the atherogenic effects of Cd.

Cd has also been shown to induce atherogenesis through stimulation of M1 macrophage polarization via JAK2/STAT3 signaling and a subsequent production of proinflammatory cytokines, including TNF-α and IL-6 [98]. In ApoE−/− mice Cd exposure promoted atherosclerosis through the alteration of gut microbiota and increased production of trimethylamine-N-Oxide that significantly contributes to macrophage M1 polarization characterized by up-regulated NLRP3 and NF-KB p65 expression and a subsequent inflammation and increased plaque formation [99].

Along with induction of proapoptotic signaling, Cd exposure also up-regulated mRNA expression of adhesion molecules ICAM-1 and VCAM-1, as well as VE-cadherin [100,101]. Cd-induced up-regulation of von Willebrand factor mRNA and protein expression though stimulation of ETS-related gene (ERG) transcription factor signaling, but not NF-κB and GATA3 signaling, may also contribute to atherogenesis. Cd exposure-induced decrease in the abundance of *Prevotella* and *Lachnoclostridium* and Cd exposure-induced increase in the abundance of *Escherichia coli_Shigella* were associated with increased circulating LPS levels and dyslipidemia, as well as systemic, hepatic, and renal inflammation [102,103].

Taken together, the existing studies demonstrated a significant association between the epidemiology of MetS and its components, including dyslipidemia and atherosclerosis, elevated blood pressure, and hypertension, as well as hyperglycemia and DM2, whereas the association between Cd exposure and obesity is still questionable. Recent laboratory findings further expanded the potential molecular mechanisms underlying the role of Cd in metabolic syndrome by unraveling the role of autophagy, pyroptosis, ferroptosis, and NLRP3 inflammasome in cell dysfunction, as well as the systemic epigenetic effects of Cd and modulation of gut microbiota in β-cell and endothelial dysfunction, as well as insulin resistance.

### 1.2. Arsenic (As)

Arsenic (As) overexposure has previously been proposed as the potential factor aggravating development of MetS and associated metabolic disorders [104]. Although first indications of the association between As exposure and MetS were obtained in Taiwan more than a decade ago, recent findings provided additional insight into this interplay. Specifically, in an As-exposed population of Bangladesh, patients with MetS were characterized by significantly higher hair, nail, and drinking water As levels, with the latter being significantly associated with the risk of MetS components (except hypertriglyceridemia), especially in women [105,106,107]. Notably, in an endemic arsenism area of Iran, the increase in %DMA and secondary methylation index (DMA/MMA), but not iAs or primary methylation index (MMA/iAs), was associated with increased MetS risk in a study of 132 individuals [108]. Doubling of urinary As levels was associated with a significant increase in MetS (HR = 1.14 (1.01, 1.29)) in a prospective cohort of 947 midlife women in the United States characterized by low-to-moderate As exposure [109]. Although urinary total As levels were associated only with increased risk of fasting hyperglycemia but not MetS, both lower level of MMA and higher concentration of DMA were related to increased risk of MetS [110].

Polymorphisms of critical genes involved in the pathogenesis of metabolic syndrome have been shown to significantly modulate sensitivity to As-induced metabolic syndrome. Specifically, dominant genetic model ADIPOQ/rs266729 and recessive genetic model FABP2/rs1799883 significantly reduced and increased the risk of hypertension in As-exposed subjects, respectively, whereas KEAP1 rs11545829 SNP mutation homozygote AA genotype reduced the association between DM2 and urinary As levels in an associate study of 699 individuals [110,111].

The existing data provide strong evidence that As exposure interferes with mechanisms involved in the pathogenesis of obesity, yet they remain inconclusive [112,113]. The most recent data suggest that particular As species may be differentially associated with obesity. In Bangladeshi adults MMA and DMA were differentially related to BMI, characterized by negative and positive association, respectively [114]. Correspondingly, despite an inverse relationship of total urinary As and MMA levels with BMI, DMA levels were found to increase along with BMI up to 4.26 μg/L/day, while at higher concentrations BMI tended to decrease [115]. Correspondingly, urinary As levels adjusted for creatinine and osmolality were inversely associated with BMI and waist-to-height ratio), in agreement with earlier observations in Taiwan [116,117]. Altogether these data support a significant role of As metabolism through methylation in its effect on obesity.

The observed associations between As exposure biomarkers and obesity seem to be mediated by As-induced adipose tissue dysfunction [118]. In an in vitro model of umbilical cord derived mesenchymal stem cells, As exposure inhibited adipogenesis through down-regulation of PPARγ, FABP4, and SLC2A4 mRNA expression and a concomitant up-regulation of proinflammatory cytokine expression. Moreover, results from an epidemiological study using 22 cord blood corroborated the observed in vitro findings, demonstrating a significant inverse correlation between toenail As levels and PPARG, aP2, and SLC2A4 mRNA expression in prenatally exposed children [119]. These findings agree with the results of earlier studies demonstrating down-regulation of PPARγ, aP2, and C/EBPs expression in inhibitory effect of As on [120,121]. Inhibition of adipogenesis upon As exposure may also involve ERS and up-regulation of CHOP10 that decreases C/EBPβ binding activity and affects adipogenic transcription factor activation [24]. Inhibitory effect of As exposure on adipogenesis was shown to be mediated by up-regulation of miR-29b expression that affected cell cycle by sustained cyclin D expression [122].

As exposure was shown to induce retroperitoneal adipose tissue hypertrophy with impairment of insulin-induced lipolysis characterized by down-regulation of genes involved in lipolysis, fatty acid uptake, oxidation, and glycerol transport [123]. In agreement with the results of epidemiological studies. demonstrating distinct association between As metabolites and obesity, AS3MT deficiency is also associated with altered metabolic profile characterized by increased body adiposity and insulin resistance [124]

The effect of As on body weight may also be mediated additional mechanisms beyond modulation of adipogenesis. As exposure in mice significantly reduced metabolic heat production as well as an increase in body fat and inguinal WAT mass due to down-regulation of protein expression of TOMM20, PGC1A, and CPT1B involved in mitochondrial biogenesis, functioning, and fatty acid oxidation, altogether being indicative of reduced energy expenditure [125]. Zuo et al. (2019) demonstrated cold intolerance, lipid accumulation in BAT, and impaired lipolysis [126]. Correspondingly, in an in vitro study reduced brown adipogenesis and down-regulation of UCP1 expression significantly contribute to inhibition of thermogenesis upon As exposure [127]. In addition, As-induced alterations in gut microbiota including reduced relative abundance of *Lactobacilli*, *Bifidobacteria*, *Akkermansia*, *Lachenospiraceae*, *Fecalibacterium*, *Eubacterium*, and clostridium coccoid group with increased Enterobacteriaceae abundance may contribute to inhibition of adipogenesis, lipolysis, and thermogenesis in gonadal WAT, as well as increased adipogenesis and thermogenesis in BAT, as well as systemic inflammation [128]. It is also worth mentioning that Gong et al. (2021) demonstrated transgenerational effect of paternal As exposure, characterized by reduced body adiposity in F2 offspring and increased susceptibility to diet-induced increase in body adiposity in F3 offspring [129]. Taken together, the potential role of As as an obesogen remains unsolved, although adipose tissue dysfunction induced by As exposure is expected to aggravate metabolic risk [130,131].

Hyperglycemia and type 2 diabetes mellitus (DM2) have also been shown to be related to As exposure. A meta-analysis of 38 studies by Sung et al. (2015) revealed a significant association between As exposure and DM with a risk ratio of 1.71 (95% CI 1.32–2.23) [132]. Recent epidemiological findings corroborated these results. Specifically, the results from NHANES (2015–2016) demonstrated that total urinary As level was directly associated with insulin resistance after adjustment for possible confounding factors [133]. In American Indian adults without prediabetes exposed to low-to-moderate doses of As, total urinary As levels were also associated with increased hazard ratio of DM2 [134]. It has been also demonstrated that hyperglycemic effect of As exposure is more profound in subjects with low skeletal muscle mass, low socioeconomic status as well as obesity may be associated with increased sensitivity to As-induced diabetes [135,136,137]. Polymorphisms of genes involved in diabetes pathogenesis, including IL8RA, TXN, NR3C2, COX5A, and GCLC, significantly modulated the association between urinary As levels and the odds of diabetes in a general Spanish population [138].

**Figure 3:**
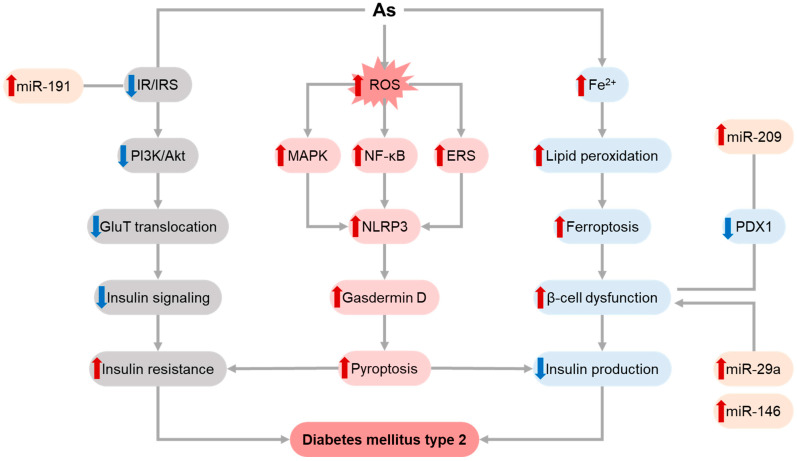
Molecular mechanisms underlying hyperglycemic effects of As exposure. As exposure was shown to induce β-cell dysfunction and death through ferroptosis, resulting in reduced insulin production. Alteration of IR/IRS/PI3K/Akt signaling pathway upon As exposure was shown to inhibit insulin signaling, thus resulting in insulin resistance. As-induced ROS overproduction was also shown to promote NF-κB and MAPK activation with subsequent NLRP3 inflammasome activation and gasdermin D-dependent pyroptosis that is considered as a potential player in the development of both insulin resistance and insulin deficiency. Activation of endoplasmic reticulum (ER) stress upon As exposure also promote inflammasome activation. In addition, recent findings demonstrated that that As-induced β-cell dysfunction may be mediated by up-regulation of miR-29a, miR-146, and miR-209 expression. The effects of the latter are at least partially associated with inhibition of transcriptional factor PDX1 expression. In addition, metalloid-induced miR-191 up-regulation significantly contributes to inhibition of IRS1/PI3K/Akt pathway. Different colors (pink, grey, blue, yellow) are indicative of distinct pathways for better visualization. Red and blue arrows show the increase and decrease effects.

The role of As exposure in DM2 pathogenesis involves modulation of carbohydrate metabolism, insulin sensitivity in various tissues, insulin production, and beta-cell dysfunction [139] (Figure 3). It has been proposed that As-induced alterations in insulin secretion mainly contribute to impaired carbohydrate metabolism rather than peripheral insulin resistance. Both organic and inorganic As species reduced glucose-induced insulin secretion in murine pancreatic islets through inhibition of Ca2+ influx [140,141]. Styblo’s group demonstrated that inorganic and organic arsenicals significantly altered Krebs cycle and mitochondrial function, and impaired amino acid, carbohydrate, phospholipid and carnitine metabolism in beta-cells, altogether resulting in inhibition of glucose-stimulated insulin secretion [142,143].

As displayed toxic effects on pancreatic beta cells through the induction of ROS overproduction and oxidative stress, as well as p38-MAPK- and NF-kB-mediated inflammation. Beta-cell damage was shown to be dependent on NLRP3 inflammasome activation through As-induced down-regulation of Annexin A1 mRNA and protein expression and endoplasmic reticulum stress with IRE1α phosphorylation, contributing to pyroptosis and β cell dysfunction [144,145]. Another mechanism of As-induced beta-cell dysfunction involves ferroptosis [146]. Dysregulation of autophagy also appears to play a significant role in As-induced beta-cell damage. Specifically, As-induced toxicity to beta cells was shown to be associated with induction of endoplasmic reticulum stress and subsequent activation of LC3II-mediated autophagy, whereas PERK knockdown ameliorated As-induced autophagy. At the same time, As exposure resulted in ROS-induced down-regulation of PPARγ signaling, resulting in inhibition of PTEN-induced kinase 1 (PINK1)-mediated mitophagy and subsequent beta-cell apoptosis [147].

The diabetogenic effect of As was shown to be mediated by epigenetic mechanisms. In particular, As-induced inhibition of insulin secretion in pancreatic beta cells is associated with altered DNA methylation of the glucose transporter 2 (Glut2) gene [148,149]. Ramdas et al. (2018) demonstrated that β-cell dysfunction in response to As treatment may be mediated by miR-2909-mediated inhibition of pancreatic duodenal homeobox 1 (PDX1) protein expression, which is considered as a key transcription factor regulating β-cell functioning [150]. Up-regulation of miR-146 following As exposure was shown to contribute to beta cell dysfunction and altered insulin synthesis [151,152]. Adverse effects of As exposure on beta-cell gene expression were also shown to be mediated by up-regulation of miR-29a [152].

In addition to alterations of insulin production, As significantly affected the insulin-signaling cascade in various tissues, including liver, skeletal muscle, and brain. Transcriptomic analysis demonstrated that As and high-fat diet coexposure significantly affects hepatic expression of genes involved in insulin signaling [153]. Both inorganic arsenite and methylarsonite significantly down-regulated Akt phosphorylation, resulting in impaired PI3K/Akt pathway signaling and subsequently leading to inhibited insulin-induced activation of glycogen synthase along with stimulating glycogen phosphorylase activity [154]. Further studies revealed potential mechanisms which mediate the adverse effects of As on insulin signaling. Specifically, in liver cells, insulin resistance following As exposure was shown to be mediated by increased mitochondrial ROS production, mtDNA oxidation, and PINK1-mediated mitophagy with subsequent NLRP3 inflammasome activation [155].

Another mechanism of As involvement with MetS may involve arsenite methyltransferase (AS3MT)-mediated N6-methyladenosine (m6A) methylation of NLRP3 mRNA and its subsequent activation [156]. Activation of NLRP3 inflammasome was shown to contribute to As-induced hepatic insulin resistance by suppression of glycolysis via binding to pyruvate kinase, liver and RBC (PKLR). As-induced NRLP3 inflammasome activation along with inhibition of gasdermin D ubiquitination resulted in promotion of gasdermin D-mediated pyroptosis and subsequent hepatic insulin resistance [157,158]. As-induced increase in miR-191 expression significantly inhibits IRS1/PI3K/Akt pathway, resulting in a reduction of GLUT4 translocation in hepatocytes [159]. In skeletal muscles, As-induced insulin resistance characterized by reduced IRS, Akt, and GluT4 protein expression was shown to be dependent on ROS-induced autophagy [160]. It has also been demonstrated that As exposure induces insulin resistance in brain cells by inhibition of IR and IRS-1 tyrosine phosphorylation, due to impaired insulin receptor tyrosine protein kinase activity and down-regulation of PI3K/Akt signaling [160,161].

As exposure plays a significant role in atherogenesis. A meta-analysis of five previously published studies demonstrated that As exposure results in reduced HDL-C and increased LDL-C concentrations [162]. It has been demonstrated that organic and inorganic As metabolites are differentially associated with lipid profile. Specifically, urinary As levels, and especially arsenic acid concentrations, were shown to be directly associated with serum TC, whereas both total As and its methylated metabolites (DMA/MMA) were characterized by a direct relationship with LDL-C [163]. It is also notable that dietary As sources also have a significant impact on the relationship between As exposure and dyslipidemia. Total As intake with dietary items and water was found to be associated with increased HDL-C levels and hypertension risk, whereas As intake from rice consumption directly correlated with LDL-C concentration [164]. In agreement with the observed association between As exposure and atherogenic lipid profile, a positive relationship between increased risk of carotid atherosclerosis as well as hypertension and drinking water As levels, even within the WHO provisional guideline value of 10 µg/L, was found [165].

Additional epidemiological studies have found an association between body As burden and the risk of dyslipidemia and atherosclerosis [166,167]. In agreement with these epidemiological data, laboratory findings demonstrated an adverse effect of As exposure on serum lipid profile, characterized by reduced HDL-C and increased circulating TC, TG, and LDL-C concentrations [168]. Increased LDL oxidation upon As exposure may be also mediated by reduced PON1 activity [169]. In addition, As exposure was shown to inhibit cholesterol efflux from macrophages via ROS-induced activation of DNA methyltransferase 1 (DNMT1) and subsequent ABCA1 promoter methylation [170].

In HUVECs, As exposure significantly up-regulated vascular cell adhesion molecule-1, intercellular adhesion molecule-1, and pentraxin 3 mRNA and protein expression, being in agreement with the observed association between As levels and circulating VCAM-1 levels and 15-F2t-IsoP in adults from the New Hampshire Health Study exposed to low-to-moderate doses of As [171,172]. As reduced fibrinolytic tissue-type plasminogen activator synthesis through the Nrf2-mediated pathway [173]. Increased expression of As3MT was observed in plaque-resident cell types, being indicative of its essential role in atherogenic effect of As [174]. As-induced dysregulation of lipid metabolism were also shown to be mediated by alterations in gut microbiota, as evidenced by the opposite effects of As on lipid metabolism in antibiotic-treated mice [175].

Epidemiological studies have provided evidence that As overexposure is associated with dysregulation of blood pressure and hypertension [32]. The results of a meta-analysis demonstrated a significant dose-dependent relationship between increased water As levels and hypertension, when every 1 µg/L increase in water As was associated with an 0.08% increase in the odds of hypertension [176]. Correspondingly, a 4.03 mmHg (95% CI: 1.24–6.82) increase in blood pressure was revealed in As-exposed subjects in another meta-analysis [177]. As accumulation in the organism was also associated with blood pressure elevation and hypertension. Specifically, hair As levels in hypertensive women were more than two-fold higher as compared to control subjects, although being unrelated to single nucleotide polymorphisms in genes related to phase II metabolism enzymes [178]. In turn, increased serum As levels promoted the association between ccm3 genetic polymorphism and elevated blood pressure [179]. Prenatal exposure, as assessed by maternal urinary As levels, was significantly associated with increased blood pressure in adolescents along with current exposure [180]. It is also notable that the As-associated elevation of blood pressure was higher in subjects with diabetes at baseline [181]. In agreement with the epidemiological studies, As exposure significantly increased blood pressure in laboratory rodents. As treatment also altered vascular relaxation induced by acetylcholine and sodium nitroprusside or 8-bromo-3′,5′-cyclic GMP [182,183,184,185].

The hypertensive effect of As would be expected to result from the imbalance between vasorelaxation and vasoconstrictor signals, especially reduced NO production or bioavailability. It has been demonstrated that As exposure resulted in blood pressure elevation in rats due to inhibition of eNOS and up-regulation of iNOS and NOX expression altogether resulting in reduced NO bioavailability [186]. The latter may be also mediated by up-regulation of asymmetric dimethylarginine (ADMA) production due to its reduced degradation by dimethylarginine dimethylaminohydrolase (DDAH), as well as depletion of L-arginine levels [187].

As has been shown to promote the vasoconstrictor effect of angiotensin II through an increase in circulating angiotensin II level and ACE activity, up-regulation of AT1 receptor and Gαq/11 protein expression, and MAPK pathway, thus promoting angiotensin II signaling [188]. Rahaman et al. (2023) demonstrated that the hypertensive effect of As in mice involves an increase in angiotensin II and a concomitant reduction in angiotensin (1–7) levels, as well as down-regulation of aortal ACE2, MasR, SIRT1, and specificity protein 1 (Sp1) expression [189]. Interestingly, endothelial dysfunction upon As exposure was shown to be mediated by autophagy activation, whereas As-induced hypertension and autophagy in aorta, characterized by increased Beclin-1 levels and LC3II/I ratio, may be dependent on the up-regulation of SIRT1 signaling [190].

Taken together, recent epidemiological studies provide evidence supporting the association between As exposure and metabolic syndrome and its components, with a special focus on its metabolism and the differential relationship between As metabolites with MetS. These studies have further deepened the understanding of the role of oxidative stress, inflammation, beta-cell dysfunction and insulin resistance, and the dysregulation of lipid metabolism and vascular tone regulation in particular components of MetS, demonstrating the role of micro RNAs, epigenetic effects, gut microbiota modulation, as well as the induction of pyroptosis and autophagy in As-induced MetS.

### 1.3. Mercury (Hg)

Humans can be exposed to Hg in three distinct forms, elemental Hg vapor (Hg^0^), inorganic mercury compounds, and organic mercury compounds. All Hg compounds can be potentially harmful to humans, depending on the dose, exposure route, and duration [191]. In respect to MetS, methylmercury (MeHg) is the major species of Hg to be investigated, although there are some reports on HgCl_2_. MeHg is a major environmental pollutant that is a contaminant of our fish supply, with the highest concentrations present in large predatory species, such as swordfish, tuna, and shark. MeHg has long been described as a neurotoxicant, with its effects varying depending on the life-stage of the individual, dose, and duration of exposure. Developmental exposure to MeHg has been linked to cognitive and behavioral dysfunction in children, while cumulative exposure to MeHg over an adult’s lifetime has been linked to the development of neurodegenerative diseases, such as Parkinson’s disease [192,193]. Early studies of MeHg poisoning demonstrated an anorexic effect on weight in humans [194,195] and experimental animals [194,196,197].

The evaluation of longitudinal data has revealed relationships between MetS, obesity, and lipid dysregulation with various types of Hg exposures. NHANES, conducted at various locations across the United States, has revealed associations between heavy metal exposure and MetS and obesity comorbidities. In examining NHANES data from ~9500 participants, from 2003–2014, Wang et al. found correlations between rates of obesity, including high BMI, higher skinfold thickness, and high percentage body fat, and comorbidities, such as type 2 diabetes, with cumulative exposures to mixtures of metals, including Hg (measured in blood and urine), As (measured in urine), Pb (measured in blood), Cd (measured in blood), Ba(measured in urine), and Tl (measured in urine) [22]. These observations were independent of age, race, cigarette smoking, education, or physical activity level. Similar associations were made in examining larger numbers of NHANES participants from 2007–2018 [198]. The Bulka et al. report of NHANES data from 2011–2014 revealed an increased odds ratio for developing obesity with pairs of metals rather than mixtures of many metals. In 1088 participants, the authors found an increased chance of obesity in individuals exposed to both Hg and Mn and in individuals exposed to Hg and As [199]. In the individuals exposed to As and Hg, there was an elevated prevalence of high blood pressure, low HDL cholesterol, and high triglycerides among those with greater exposures [199]. Similarly, in participants from the Korean National Environmental Health Survey (KoNEHS) from 2015–2017, increased blood and urine levels of Hg, Pb, and Cd were associated with obesity and nonalcoholic fatty liver disease [200]. Conversely, in the Study of Women’s Health Across the Nation Multi-Pollutant Study with 947 midlife women, mixtures of Cd, cesium, Hg, molybdenum, Pb, and tin did not associate with MetS markers (high blood pressure, impaired fasting glucose, abdominal obesity, high triglycerides, and poor high-density lipoprotein cholesterol) [109].

While these data explored exposure to heavy metal mixtures, epidemiological data has emerged linking exposure solely to MeHg and MetS parameters. Lee found that elevated blood Hg levels were associated with higher body weights and obesity in participants from KoNEHS from 2011–2013 [201]. Elevated blood Hg levels were also found to correlate to hyperlipidemia and increased serum liver enzymes, a marker of liver disease, in participants from KoNES from 2012–2014 [202]. In these studies, Hg exposure occurred through the dietary consumption of MeHg in fish and seafood. Likewise, high blood Hg levels were observed in obese individuals with high body mass index, waist circumference, and visceral adipose tissue; high blood pressure, fasting glucose, and insulin resistance after adjusting for alcohol and cigarette usage [20]. Additionally, links between Hg exposure and type 2 diabetes were found upon examining 646 participants from the National Nutrition and Health Survey in Taiwan (NAHSIT) 2005–2008 [203]. While blood Hg levels inform about current exposure to Hg, long term exposures to Hg are better reflected by hair or nail accumulation, two biomarkers that grow, and have Hg deposited into them, at defined rates [204,205]. In a study of 500 participants, the Trace Element Study of Korean Adults, higher toenail Hg levels were associated with regular consumption of large predatory fish and mammal meat, as well as the presence of MetS [206,207]. Furthermore, individuals with higher toenail selenium, as reflected in diet, had lower Hg levels and MetS [206]. Similarly, in a study of 440 adults, increased hair Hg levels in overweight and obese individuals were associated with adverse metabolic markers, such as elevated serum creatinine, uric acid, glucose, LDL, and triglycerides, as compared to individuals with high hair Hg and normal body weight [208].

It has long been appreciated that children and the developing fetus are at higher risk for the neurotoxicity associated with MeHg exposure [209]. As rates of childhood and adolescent obesity are on the rise globally [210,211], it is critical to assess whether childhood obesity is influenced by Hg. Emerging data suggest that early life exposure to Hg, either in utero or during childhood, can influence development of obesity and MetS in children and adolescents. Women with high blood levels of nonessential metals, including As, Ba, Cd, Cs, Pb, and Hg, during their first trimester of pregnancy had children with larger trunk fat mass index, waist circumference, and BMI in mid-childhood and early adolescence [212]. These data suggest that early exposure to metals during pregnancy may affect the weight and development of obesity in children many years post-birth. Similar results were found in a study of 1442 mother–child pairs that examined the levels of only Hg, Pb, and Cd [213].

In a small study of 92 mother–child pairs (all children 6 years of age) conducted in New Zealand, the level of Hg measured in mothers’ hair correlated to the level of Hg in the hair of their children, suggesting a common source of Hg, i.e., a fish-based diet [214]. Interestingly, the incidence of obesity was significantly associated with higher levels of Hg in the children, but not in the mothers [214], suggesting Hg had an effect on development in children, leading to higher body weights, than similar levels of Hg in the mother. In utero exposure to low maternal blood Hg (1.04–3.7 μg/L) increased the risk of developing childhood overweight or obesity between ages 2–15 years [215]. The risk was augmented if the mother was diabetic, overweight, or obese [215], suggesting that the Hg might interact with other obesogenic factors circulating in the mother’s blood. Interestingly, adequate maternal folate levels could mitigate the effects of blood Hg on developing overweight or obesity in childhood as compared to mothers with insufficient folate [215].

An examination of the KoNEHS from 2010–2013 identified that high levels of total blood Hg in adolescents was associated with increased body weight, abdominal obesity, high waist-to-height ratio, and an increased risk of developing obesity [216,217]. Furthermore, upon combining data from 2010–2013 and 2016 KoNEHS, it was found that blood Hg was higher in males than in females, and male adolescents with the highest levels of blood Hg were at a higher risk of hypercholesterolemia, independent of body weight [218]. This agrees with data from 5400 children and adolescent participants in NHANES from 2011–2014, where blood Hg levels correlated with serum cholesterol levels [219]. While information exists on Hg’s association with childhood and adolescent Hg, whether these children remain obese into adulthood has only recently been investigated. Betanzos-Robledo et al. reported on data from the Early Life Exposures in Mexico to Environmental Toxicants study, where 100 adolescents (age 14–16 years) were recruited, measuring BMI, blood Hg, and blood Pb levels, then at age 21–22 years, fat accumulation measurements were performed. High blood Hg levels in adolescence were associated with increased subcutaneous and abdominal fat stores in early adulthood [220]. It will be interesting to pursue this population in the future to reevaluate Hg levels and fat accumulation as the participants age.

From many of these epidemiological studies, it is apparent that in higher weight individuals and patients with MetS, there are elevated levels of Hg in blood, nails, and hair. There are two ways to interpret these data: (1) Exposure to Hg can influence the development of MetS; or (2) Individuals with higher body weight have altered toxicokinetics, resulting in less elimination of Hg and the retention of higher levels of Hg than in lower body weight individuals. Recent toxicokinetic analysis in participants that consumed known concentrations of MeHg in tuna steaks revealed no statistical significance between normal body weight, overweight, or obese individuals in the elimination rate of Hg from hair [221]. This suggests that MeHg may influence the development of MetS rather than body weight altering MeHg toxicokinetics. Mechanistic studies in experimental animal models and cell culture have been performed that build a case that exposure to MeHg can cause lipid dysregulation and the physiological changes associated with the development of MetS.

The effect of MeHg on bodyweight in rodents appears to be dose-dependent. High doses of MeHg (5 mg/kg/day for 30 days) in Wistar rats were shown to cause significant weight loss [222]. Similar doses in C57BL/6J mice and KK-Ay genetically obese mice had anorexic effects [223,224]. Rats that were exposed to lower doses of MeHg (1 or 3 mg/kg/day) did not have significant changes in total body weight [222]. It should be pointed out that in many rodent models of both obesogen and diet induced obesity, increased body weight is not always observed, while other parameters, such as serum lipids, adipose composition, and hormone levels might differ between test and control rodents [225]. KK-Ay diabetic mice accumulate more Hg than non-obese mice and show enhanced neurological damage and inflammation compared to non-obese mice [226]. There also appear to be gender differential responses to MeHg in C57BL/6J mice. In males exposed to 5 ppm MeHg for 30 days, ghrelin (hunger hormone) was increased as compared to control mice, and led to body weight gain by activating the AMP-activated Kinase (AMPK)/Uncoupled protein 2 (UCP2) signaling pathway. However, ghrelin pathways were inhibited in female mice exposed to the same regimen as the male mice [226]. Furthermore, there were sex differences in neuropeptides pro-omiomelanocortin (Pomc), orexigenic peptide Agouti-related peptide (Agrp), and leptin [197,224].

MeHg has been shown to alter serum lipid and glucose profiles in rodents, similar to type 2 diabetes and dyslipidemia characteristic of MetS. Mice exposed to 40 μg/mL MeHg in drinking water for 2 weeks had adverse effects on their serum lipid profiles as compared to the non-exposed mice [227]. MeHg elevated total cholesterol levels as well as non-high density lipoprotein (non-HDL) levels [227], markers of dyslipidemia and risk factors for obesity and cardiovascular diseases. Mice exposed to lower doses of MeHg in drinking water (2 or 20 mg/L) for 30 days had increased body weight as compared to non-exposed mice, as well as elevated serum triglycerides, total cholesterol, as well as markers of systemic inflammation and oxidative stress [228,229,230]. Similar elevations in triglycerides and cholesterol were observed in Nile Tilapia (*Oreochromis niloticus*) and Bluegill (*Lepomis macrochirus*) exposed to MeHg [231,232], suggesting the effects of MeHg on circulating lipids are conserved across species. Mice exposed to 2.5, 5, or 10 mg MeHg/kg/day for 4 weeks showed glucose intolerance, insulin resistance, and hyperglycemia [233]. MeHg significantly increased ROS, leading to lipid peroxidation in the pancreatic islets and decreased thiol and ferric reducing antioxidants, while upregulating caspases, leading to apoptosis [233]. These data suggest that MeHg can induce changes in the pancreas similar to type 2 diabetes associated with MetS.

Models of MeHg exposure to adipocyte and islet cell lines, as well as the organism *Caenorhabditis elegans* have provided mechanistic links on MeHg’s propensity to induce metabolic dysregulation. MeHg has been shown to increase markers of oxidative stress, such as lipid peroxidation product 4-hydroxynonenal, following an acute treatment in 3T3-L1 adipocytes as well as alter cellular morphology and lipid content [234]. MeHg treated 3T3-L1 increased release of adiponectin and resistin [234], a hormone released by adipocytes that regulates blood glucose levels and inflammation [235]. In obese individuals, higher levels of resistin in serum were noted [235], similar to MeHg exposure to 3T3-L1 cells. The alterations in adipocyte phenotype, lipid peroxidation, and hormone secretions in response to MeHg were attenuated by the antioxidant theaflavin-3,3′-digallate [234], suggesting an important role for oxidative stress in the development of MeHg-induced metabolic dysfunction in vitro. In a model of 3T3-L1 adipocyte differentiation where MeHg was continually supplied for 8 days, MeHg enhanced the development of the lipid droplet, accumulating more triglycerides in MeHg-exposed cells than in untreated cells [236]. Furthermore, markers of mature adipocyte differentiation were significantly increased in the MeHg-treated cells, which included expression of PPARγ, adiponectin, and fatty acid binding protein [236]. MeHg was also shown to increase autophagy markers in this study. Interestingly, treatment of 3T3-L1 cells early in development with an autophagy inhibitor chloroquine prevented the effects of MeHg on the lipid droplet; however, chloroquine had no effect on MeHg-induced alterations if the cells received the inhibitor during late stages of development [236]. This suggests that early induction of autophagy in pre-adipocytes can drive the differentiation into mature adipocytes in response to MeHg. The effects of MeHg on driving adipocyte differentiation seems to be conserved across species. In primary pre-adipocytes from rainbow trout (*Oncorhynchus mykiss*) exposed to MeHg for 6 days, there was an accumulation of neutral lipids as well as the upregulation of genes that are markers of mature adipocytes, including perilipin 2, apolipoprotein Eb, fatty acid synthase, and fatty acid binding protein [237].

The differentiation of pre-adipocytes into mature adipocytes is a complex process controlled by multiple transcription factors working in concert to express the proteins necessary for the function of storing lipids and endocrine signaling. The differentiation of pre-adipocyte 3T3-L1 cells into mature adipocytes involves early induction of C/EBPβ transcription factor, which transactivates the expression of C/EBPα, PPARγ, and SREBP1 transcription factors, allowing for the expression of genes that are important for lipid storage and mobilization [238]. MeHg treatment of *Caenorhabditis elegans* has been shown to increase triglyceride content and lipid storage sites, as well as increase the expression of lipid storage, mobilization, and synthetic genes [239,240]. Interestingly, the effects of MeHg on lipid dysregulation could be modified by the bacterial diet fed to the worms [240], suggesting that dietary factors that the worms are exposed to at the same time as MeHg can affect the lipid dysregulation phenotype. MeHg-dependent lipid dysregulation in worms was associated with increased expression of *cebp-1* (homolog to human C/EBP), *nhr-49* (homolog to PPARγ), and *sbp-1* (homolog to SREBP1) transcription factors [239]. Genetic ablation of *cebp-1* prevented the MeHg-induced increases in triglyceride levels, lipid storage, and up-regulation of lipid metabolic genes [239]. These data demonstrate the potential for MeHg to activate important regulators of lipid metabolism, and provide evidence that MeHg may alter the adipocyte differentiation process through similar mechanisms in mammalian systems.

In addition to transcription factor activation required for adipocyte differentiation, it is apparent that expression of microRNAs (miRNAs) are important phenotypic regulators involved in the development of MetS. MiRNAs are short, non-coding RNAs which repress the expression of target genes by base pairing with the 3′ UTR region of the gene’s mRNA, leading to translational repression [241]. Multiple studies have shown that miRNA expression is altered in the adipose tissue of individuals with metabolic disorders [241,242]. Altering the ability of *C. elegans* to express miRNA significantly affected the ability of MeHg to cause lipid dysregulation [243]. This suggests that MeHg may affect the expression of the miRNA sequences involved in lipid homeostasis. Further characterization of the expression of adipogenic or anti-adipogenic miRNA sequences in response to MeHg is needed to fully understand the mechanisms of MeHg-induced adipogenesis.

MeHg has been shown to reduce the viability of the HIT-T15 pancreatic cell line and primary mouse pancreatic islets and to decrease the amount of insulin secreted by these cells [244]. In HIT-T15 cells, MeHg induced apoptosis, disrupting the mitochondrial membrane potential with release of cytochrome c, and activated caspases [244]. MeHg-induced apoptosis in β-cells was shown to be dependent on oxidative stress, as the antioxidant N-acetylcysteine prevented the β-cell apoptosis [244]. Similarly, MeHg decreased the viability and insulin production in the RIN-m5F cell line, displaying not only markers of mitochondria-dependent apoptosis, but also of endoplasmic reticulum (ER) stress [245]. Both the mitochondrial and ER stresses were shown to be dependent on ROS generation and c-Jun N-terminal kinase (JNK) activation, as treatment with N-acetylcysteine, 6-hydroxy-2,5,7,8-tetramethylchroman-2-carboxylic acid (trolox), or a specific JNK inhibitor (SP600125) prevented MeHg-induced toxicity to the RIN-m5F cells [245]. These data suggest that the glucose intolerance and low insulin levels observed in vivo may be due to the cytotoxicity of MeHg to pancreatic β-cells.

In contrast to MeHg, inorganic HgCl_2_ has been shown to be anorexic in both rodents and cell lines. Male Wistar rats exposed to a low dose of HgCl_2_ for 60 days (0.07 μg/kg/day) showed a decrease in the size of epididymal white adipocytes but had increased serum triglycerides, decreased insulin, and increased plasma glucose levels as compared to unexposed rats [246]. Furthermore, important adipocyte regulators such as PPARα, and PPARγ mRNA were increased, suggesting impaired adipocyte function [246]. Similarly, Kawakami et al. found that HgCl_2_ exposure to mice fed a high fat diet, decreased visceral white adipose tissue size and decreased leptin (the satiety hormone) and insulin, but also decreased PPARα and PPARγ mRNA [247], suggesting there might be differential responses in different populations of adipocytes. Similarly, in 3T3-L1 and C3H10T1/2 adipocyte cell lines, HgCl_2_ decreased the number of phenotypic adipocytes without affecting cell viability [248], suggesting the HgCl_2_ prevented adipocyte differentiation. Markers of differentiation, such as expression of PPARγ and GLUT4, as well as lipid storage (measured by Oil Red O staining), were significantly reduced in HgCl_2_-treated cells than in the control adipocytes [248].

Overall, epidemiological studies, in vivo animal studies, and in vitro studies have described the potential of MeHg to cause the phenotypic changes characteristic of MetS. Further research is needed to define the mechanisms of MeHg-induced metabolic dysfunction as well as to better understand the exposure conditions that can cause metabolic dysregulations in humans.

### 1.4. Lead (Pb)

Lead (Pb) is a well-established toxic heavy metal that is present in ecosystems due to its long-standing use in many industrial applications in the past, such as gasoline and paints. Currently, most of the past processes that required the use of Pb have been restricted to reduce its release into the environment and reduce exposure to humans in at least major portions of the world [249]. Although acute intoxications with high doses of Pb are infrequent at present, exposure to environmental concentrations of this metal still comprise a public health issue. A notable exception is the recent poisoning incident in Flint, Michigan, USA, in which Pb contamination of the drinking water supply increased the percentage of children with elevated blood Pb levels (BLL) [250]. As children are the most vulnerable population, the Center for Disease Control and Prevention (CDC) uses 3.5 g/dL as the blood lead reference value (BLRV) for children as a level of concern to take action. Having no known biological function, Pb harms developing organisms primarily through the central nervous system [251,252]. Notably, increasing evidence demonstrates that other systems are affected by Pb as well, such as renal, cardiovascular, hematopoietic, reproductive, and endocrine, including the disruption of metabolic pathways in a variety of tissues.

Recently, Pb exposure has been related to the development of MetS and obesity. Based on the results of a study on 2833 Korean subjects (1230 men and 1603 women), BLL were significantly correlated with all metabolic syndrome variables, including systolic and diastolic blood pressure, waist circumference, fasting blood sugar, TG, and HDL cholesterol. [253]. Another study included 3787 adults (aged ≥19 years) who participated in the Korean National Environmental Health Survey 2015–2017 and investigated the association of toxic heavy metals (Pb, Hg, and Cd) with metabolic disorders. In this case, both Pb and Hg exposures were associated with an increased risk of obesity. In contrast, no correlation was found in a male population (313 men aged 50–75 years) in north-western Poland between BLL (median of 77 μg/L) and metabolic syndrome development or exacerbation [254]. Interestingly, analysis of the data from NHANES 1999–2006 supported an inverse association of BLL and body weight outcomes in children and adolescents and adults in the U.S. population. In children, adolescents, and adults, a lower BMI was associated with higher BLL quartiles (1.1 to 1.6 μg/dL or higher) [255].

Similarly, rat and mouse model studies have linked Pb with MetS. A study conducted by Wang and colleagues [256] revealed that chronic Pb exposure to mice to 200 mg/L Pb or/and HFD for 24 weeks resulted in 10 μg/dL in the blood Pb content of rodents, significantly increased body weight, visceral obesity, fasting blood glucose levels, and insulin resistance. In addition, aggravated liver damage, hepatic lipid accumulation, and steatosis were found in HFD-fed C57BL/6J male mice. However, the effects of Pb exposure in normal diet-fed mice were not found. Further analysis showed that Pb significantly inhibited insulin signaling pathway PI3K/AKT and fatty acid β-oxidation, and accelerated fatty acid synthesis. Moreover, Pb exacerbated HFD-induced disruption of gut microbiota homeostasis. They also showed that both “HFD” only and “Pb plus HFD” animals had an increased hepatic expression of SREBP-1, FASN, and SCD-1 compared to controls, Pb, or HFD intervention alone. Furthermore, the expression of key lipolytic regulator PPAR-α and its downstream genes (CPT-1a and Acox) was downregulated, with the lowest levels noted in the “Pb plus HFD” combined group [256].

Moreover, Pb exposure has been associated with impaired glucose homeostasis. When female ZDF fa/fa obese rats were treated with 0.05% *w*/*v* Pb in drinking water, Pb exposure induced fasting hyperglycemia after 8 weeks and glucose intolerance after 12 weeks of exposure. In addition, Pb-exposed animals showed elevated hepatic triglyceride levels and increased expression of the gluconeogenic genes PEPCK and glucose-6-phosphatase [257]. In addition, in a study conducted in male Wistar rats under a similar exposure paradigm (0.05% Pb, drinking water, or fed with an HFD for 28 weeks), the animals exposed to Pb displayed impaired glucose homeostasis, as evidenced by increased fasting plasma glucose and hepatic glucose production. Moreover, the gene expression levels of PEPCK, G6PC, and FBP1 were increased. In addition, the mRNA expression levels of PEPCK, G6PC, and FBP1 in the human hepatocarcinoma HepG2 cell line were also increased in response to Pb exposure (2.5 to 10 μM Pb acetate). Interestingly, sub-chronic Pb exposure (0.2% Pb, drinking water, 32 days) was able to disrupt the insulin secretory function of Islets of Langerhans through activating GSK-3 and ER stress, and increased activity of gluconeogenic enzymes, PEPCK, and G6P in the liver, demonstrated by glucose intolerance in male adult Wistar rats [258].

## 2. Concluding Remarks

MetS is an important public health concern that impacts millions of individuals around the world. Growing evidence suggests the involvement of metals such as Cd, Hg, and Pb, and metalloids such as As, in the development of obesity, hypertension, atherosclerosis, and diabetes, suggesting that those environmental contaminants are risk factors for MetS.

**Figure 4:**
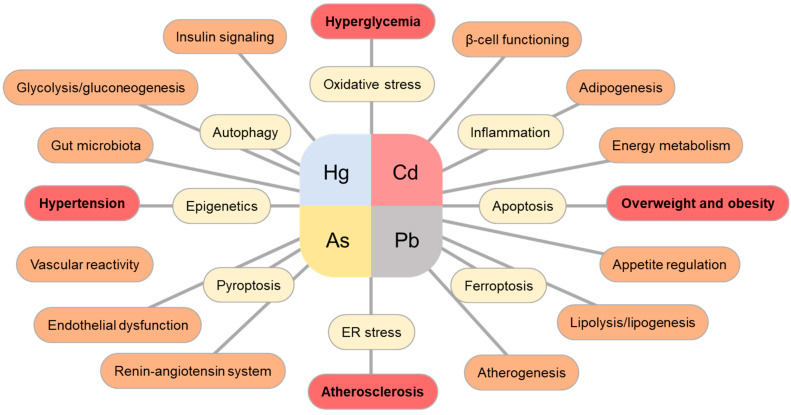
The potential mechanisms (yellow) and targets (orange) of the role of toxic metals in metabolic syndrome and its components (red). Toxic metal exposure results in induction of oxidative and endoplasmic reticulum stress, inflammation, apoptosis, as well as ferroptosis, pyroptosis, impaired autophagy, and epigenetic effects in target tissues. These mechanisms were shown to underlie the impact of heavy metal toxicity on adipogenesis, β-cell functioning, insulin signaling, appetite regulation, carbohydrate and lipid metabolism, atherogenesis, endothelial dysfunction, vascular reactivity, and gut microbiota.

Overall, epidemiological studies suggest an association between exposure to heavy metals or metalloids and MetS. Recent findings demonstrated that along with oxidative and endoplasmic stress, inflammation, and apoptosis, other novel mechanisms including ferroptosis, pyroptosis, epigenetics, and gut microbiota modulation may contribute to target tissue dysfunction during MetS pathogenesis (Figure 4).

However, a clear understanding of the mechanisms triggered by toxic compounds and MetS etiology has yet to be fully characterized. Thus, proteomic and transcriptomic analysis, alongside novel bioinformatic tools, will be crucial for enhancing knowledge of the disease mechanisms induced by toxic metals and metalloids. Moreover, studies to investigate potential molecular targets for these illnesses are urgently necessary.

## Data Availability

Not applicable.
